# The role of temperature in vitality and survival assessments of beam-trawled and discarded European plaice *(Pleuronectes platessa)*

**DOI:** 10.1093/conphys/coae036

**Published:** 2024-06-13

**Authors:** Sven Sebastian Uhlmann, Silvia Paoletti, Bart Ampe, Konstantinos Theodoridis, Marc Kochzius, Barbara Koeck

**Affiliations:** Flanders Research Institute for Agriculture, Fisheries and Food (ILVO), Animal Sciences Unit, Fisheries and Aquatic Production, Jacobsenstraat 1, 8400 Ostend, Belgium; Marine Biology - Ecology, Evolution & Genetics, Vrije Universiteit Brussel (VUB), Pleinlaan 2, 1050 Brussels, Belgium; Flanders Research Institute for Agriculture, Fisheries and Food (ILVO), Animal Sciences Unit, Fisheries and Aquatic Production, Jacobsenstraat 1, 8400 Ostend, Belgium; Marine Biology - Ecology, Evolution & Genetics, Vrije Universiteit Brussel (VUB), Pleinlaan 2, 1050 Brussels, Belgium; Royal Belgian Institute of Natural Sciences, Operational Directorate Natural Environment (OD Nature), Rue Vautier 29, 1000 Brussels, Belgium; Flanders Research Institute for Agriculture, Fisheries and Food (ILVO), Animal Sciences Unit, Fisheries and Aquatic Production, Jacobsenstraat 1, 8400 Ostend, Belgium; Marine Biology - Ecology, Evolution & Genetics, Vrije Universiteit Brussel (VUB), Pleinlaan 2, 1050 Brussels, Belgium; Fisheries Research Institute, Hellenic Agricultural Organisation - Demeter, INALE Nea Peramos, Kavala 64007; Marine Biology - Ecology, Evolution & Genetics, Vrije Universiteit Brussel (VUB), Pleinlaan 2, 1050 Brussels, Belgium; School of Biodiversity, One Health & Veterinary Medicine, College of Medical, Veterinary and Life Sciences, University of Glasgow, Graham Kerr Building, Glasgow, G12 8QQ, UK; WasserCluster Lunz Biologische Station, Inter-university Center for Aquatic Ecosystem Research, Dr. Kupelwieser-Prom. 5, 3293 Lunz am See, Austria

**Keywords:** Animal reflexes, discard survival, poikilothermic, temperature tolerance

## Abstract

Thermal stress can influence the recovery of fish released after capture. Vitality assessments using reflex and behavioural responses require that responses can be observed reliably, independent of temperature. Here, we tested whether reflex and behavioural impairment and survival of beam-trawled and discarded European plaice (*Pleuronectes platessa*) are independent from seasonal air and water temperature deviations. In total, 324 beam-trawled plaice (*n* = 196 in summer and *n* = 128 in winter) were exposed to two air temperature treatments and two water treatments (i.e. modified and ambient temperatures for both). The modified treatments (i.e. cooled in summer, warmed in winter) represent the thermal shock a fish may experience when being returned to the water. All reflexes and tested behaviours were affected by ambient temperature, with high impairment noted in summer. None of the reflexes were affected by temperature shocks alone, only body flex was. Body flex was highly impaired under every exposure combination. Fish size and duration of air exposure further influenced impairment of reflexes such as head complex and tail grab. More generally, post-release survival was assessed as 21% [95% CI: 16–28%] in summer and 99% [97–100%] in winter. Beam trawling in summer is likely to induce high reflex impairment and mortality in discarded plaice, and therefore spatial–temporal mitigation approaches should be prioritized over control of on-board temperatures.

## Teaser

In the present study, we assessed reflex impairment and its relation to survival of European plaice after capture with commercial beam-trawl fishing gear and subsequent release. We paid special attention to the effect of temperature. This may help to develop catch welfare indicators used to measure the stress that fish may experience during catch and eventual release.

## Introduction

For ectothermic marine fish, water temperature is a known factor in controlling metabolism and physiological processes (Fry *et al.,* 1947; Guderley, 2004). Environmental temperature and changes in temperature can act as a stressor for fish (Schulte, 2014) and thus can therefore exacerbate other known fisheries-related stressors (e.g. air exposure) and contribute to post-release mortality (Uhlmann *et al.,* 2016; van Der Reijden *et al.,* 2017; Kraak *et al.,* 2019; Uhlmann *et al.,* 2021; Brownscombe *et al.,* 2022). Temperature influences the fish’s response to exhaustive exercise and external stressors such as capture (Kieffer, 2000; Gale *et al.,* 2013). Understanding the role of temperature is useful in fisheries management situations where a release allowance is linked to the likelihood of post-release survival. Understanding fish thermal tolerance (often age-, size- and species-specific) is therefore particularly important when studying post-catch recovery and the likelihood of post-release survival. External environmental (ambient) temperatures, at a potentially age- and size-specific species’ critical tolerance limit, can differentially affect vital functions and recovery potential as well as other processes linked to what a fish experiences whilst being caught and post-release (Kieffer, 2000; Gale *et al.,* 2013; Brownscombe *et al.,* 2022).

Severe thermal stress (hot and cold) causes central nervous system (CNS) dysfunction in fish, with possible effects on the neural networks in the spinal cord that control locomotion (Robertson, 2004). After a thermal shock, a stimulus to a muscle may not result in a reflex response as part of a central pattern generator pathway (Grillner, 1996). Reflex robustness is commonly tested as an indication of neural integrity or health. For over a decade, researchers have been developing whole-animal welfare indicators such as vitality assessments based on non-invasive, time- and cost-efficient *in situ* proxies to help predict an animal’s post-release fate (Davis, 2007, 2010; Raby *et al.,* 2012). These proxies consist in measuring an animal’s responsiveness to innate reflexes or its evolutionarily adapted reflexive behaviours in response to external stimuli. A reflex is a rapid involuntary movement induced by a peripheral, tactile stimulus, whilst a reflexive behaviour is a more complex fixed-action pattern triggered by a cue. Both can be used within the Reflex Action Mortality Predictor framework (RAMP; Davis and Ottmar, 2006) to predict animal survival, and are referred to hereafter as reflex behaviours. Ideally, vitality assessments of captured and released fish would require testing for reflex behaviours that are context-independent of sex, size, motivation (Stoner, 2012) and ambient temperature. To what extent ambient temperatures and/or acute temperature changes (i.e. thermal shocks) can alter responsiveness of some reflexes has only been explicitly tested in a few species (Brownscombe *et al.,* 2014; Pinder *et al.,* 2019; Brownscombe *et al.,* 2022).

**Figure 1:**
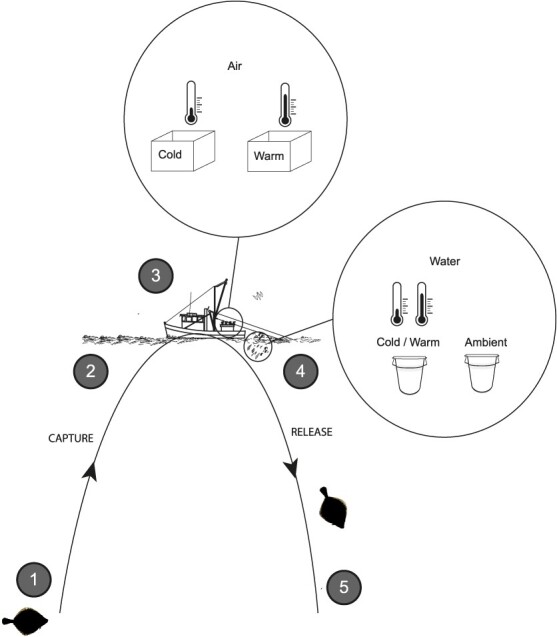
Schematic representation of the temperature treatments recreated to represent the potential seasonal temperature differences a plaice may experience during the entire experience from catch to discard. From the seafloor, the environment to which it is acclimatized (1), the plaice is hauled through the water column to the surface (2), lifted out of the water and sorted on deck whilst being exposed to air (3). To simulate air temperature shock, experimental plaice were exposed to either cold or warm air temperatures for 10 min at the ranges that were displayed in Fig. 2. During release a plaice is released at the surface (4) and swims potentially through a thermocline to the seafloor to recover (5). To simulate water temperature shock, plaice were exposed to either ambient or modified water temperatures for 10 min at the ranges displayed in Fig. 2. Table 1 returns the seasonal average (± SE) temperatures recorded across trips that represent each phase. The modified water temperature treatments depended upon the season and represented a thermocline, summarized as the following temperature conditions: 1. Summer—Modified water treatment: cold shock, presence of a thermocline upon discarding and subsequent return to the colder seafloor environment; 2. Summer—Ambient water treatment: no shock, no thermocline; 3. Winter—Modified water treatment: heat shock, presence of a thermocline upon discarding and subsequent return to the warmer seafloor environment; and 4. Winter—Ambient water treatment: no shock, no thermocline.

Following a pathway from capture to handling onboard, a fish experiences several stressors. During trawling, captured fish are caught in the net and pulled from the seafloor to the surface through the water column (Phase 1 and 2; Fig. 1). Once aboard the vessel, fish and other captured organisms are sorted under anoxic conditions, experience possibly air temperature shocks and are handled by fishers (Phase 3; Fig. 1). Upon discarding, the fish swim from the surface through the water column back to the seafloor (~10 min depending on depth) (Phase 4 and 5; Fig. 1). Depending on the season, surface water may have a different temperature (thermocline) than the ambient seafloor water temperature. In previous studies of European plaice (*Pleuronectes platessa*), a commonly discarded flatfish, thermal shocks experienced on deck were not proven significant; however, ambient (water) temperature appeared to play a role in post-release survival of plaice, with higher survival during winter (Van Beek *et al.,* 1990; Uhlmann *et al.,* 2016; Uhlmann *et al.,* 2021).

The aim of the present study was to investigate the role of temperature on vitality and probability of survival in beam-trawled and discarded plaice. In particular we assessed whether reflex impairment level and post-release survival were affected by (1) the ambient water temperature to which fish were acclimatized on the seafloor (related to fishing season); and/or (2) the air and water temperature changes (i.e. thermal shocks) that a fish may experience during trawling, sorting and discarding (potential exposure to a thermocline). This was tested on board a research vessel using experimentally induced air and water temperature deviations from seasonal seafloor water temperature. This experimental setup simulated the potential combination in sequence of cold and heat shocks that a fish may experience during the sequential experience of catch, sorting, discard and return to the seafloor.

## Materials and Methods

Five day trips were done with the R/V *Simon Stevin* in coastal waters of the Southern North Sea (ICES subdivision 4c), close to Ostend, Belgium: three trips in July 2017 (summer) and two in March 2018 (winter) at seafloor water temperatures of 20.01 ± 0.03°C and 4.64 ± 0.09°C (mean ± SE; Table 1), respectively. During each trip, beam-trawl gear was deployed three to five times (33.0 ± 0.34 min gear deployment duration, mean ± SE; Table 1). The beam trawl consisted of a 32 mm, diamond-shaped codend attached to a 3 m beam. From each deployment, 20 undersized (<27 cm, minimum conservation reference size, MCRS) plaice were randomly picked from the catch during the sorting process (12.0 ± 0.15 min, mean ± SE; Table 1) and placed into 35 L, dry PVC baskets in 4 batches of 5 individuals each. Gear deployment duration, sorting duration and total air exposure (treatment, sorting and waiting times preceding temperature exposure treatment) were standardized as much as possible across trips and deployments to minimize their relative effect on reflex impairment and survival.

**Table 1:** Summary of mean ± SD of sampling, environmental and biological variables collected during each trip, and corresponding air (cold or warm) and water (ambient or modified) temperature treatments applied to sampled plaice

Variable	Trip 1	Trip 2	Trip 3	Trip 4	Trip 5
Season	Summer	Summer	Summer	Winter	Winter
Date	7 July 2017	14 July 2017	20 July 2017	9 March 2018	16 March 2018
Total no. of deployments	3	3	4	5	3
No. plaice sampled	60	60	76	68	60
Total length (TL, cm)	15.64 ± 4.13	17.45 ± 3.97	15.59 ± 4.95	19.25 ± 7.23	19.736 ± 4.81
Gear deployment duration (min)	30.32 ± 0.47	39.33 ± 0.95	28.90 ± 6.45	32.85 ± 1.92	38.00 ± 0.95
Sorting duration (min)	9.32 ± 1.32	16.00 ± 2.18	9.02 ± 1.62	6.91 ± 3.50	6.33 ± 1.26
Total air exposure (min)	12.08 ± 5.11	16.67 ± 13.76	13.13 ± 5.99	11.16 ± 6.25	13.67 ± 5.66
Ambient air temperature (°C)	19.6 ± 0.31	17.8 ± 0.61	20.6 ± 0.66	7.3 ± 1.34	8.05 ± 0.71
124-l monitoring containers (°C)	20.66 ± 0.31	19.80 ± 0.23	19.6 ± 0.25	6.24 ± 0.9	6.8 ± 0.4
Water temperature at the seafloor (°C)^1^	19.68 ± 0.31	19.69 ± 0.02	20.51 ± 0.01	3.65 ± 0.09	5.77 ± 0.21
Water temperature at the surface (°C)^2^	19.68 ± 0.48	19.53 ± 0.05	20.48 ± 0.21	5.07 ± 0.48	8.58 ± 0.33
Cold air treatment temperature (°C)^3^	11.39 ± 0.54	14.83 ± 0.38	16.46 ± 0.47	6.09 ± 0.63	6.58 ± 1.36
Warm air treatment temperature (°C)^3^	27.50 ± 0.85	25.50 ± 0.42	26.31 ± 1.38	12.88 ± 1.60	15.16 ± 1.06
Ambient water temperature treatment (°C)^4^	19.68 ± 0.48	19.53 ± 0.05	20.48 ± 0.21	5.07 ± 0.48	8.58 ± 0.33
Modified water treatment temperature (°C)^4^	11.03 ± 0.84	16.31 ± 0.83	16.44 ± 0.62	10.88 ± 0.62	12.68 ± 0.68

Corresponding with ^1^ Phase 1, ^2^ Phase 2, ^3^ Phase 3, ^4^ Phase 4 (see Fig. 1).

### Ethics statement

This research was approved by the Animal Ethics Committee of Flanders Research Institute for Agriculture, Fisheries and Food (ILVO, Ref. no. 2017/289) and was done in accordance with European laws on protection of animals used for scientific purposes (EU Directive 2010/63/EU). This study involved an unprotected, commercially targeted flatfish species. The temperature exposure treatments were designed to simulate conventional fishing practises within the natural tolerance limits of plaice.

### Experimental design

When plaice are sorted post-catch they are held on a temperature-controlled deck that can be heated in the winter and cooled in summer resulting in plaice experiencing both cold and heat shocks during summer and winter months (Phase 3; Fig. 1; Table 1). To simulate this sorting environment, plaice were exposed to a heated or cooled air treatment. During either winter or summer, released plaice swim through a thermocline before reaching the seafloor (at the seafloor, seawater can be warmer in winter and cooler in summer) (Phase 4 and 5; Fig. 1; Table 1). To simulate this, plaice were exposed to either heated or cooled water. Exposure periods of 10 min were chosen to stay within bounds of conventional sorting times on deck as well as the time required for plaice to swim back to the seafloor after discarding. All treatment temperatures were manipulated to achieve a ~5°C difference from the ambient seafloor temperature.

The temperature difference experienced by a fish during each treatment was calculated as the absolute difference between ambient seafloor temperature and the temperature of the air or water treatments to which it was sequentially exposed (Table 2). The type of such temperature difference determined whether the shock was classified as ‘heat’ or ‘cold’ (Table 2). For the exposure to water treatments, cold shocks were only found in summer and heat shocks only in winter, as one of the two treatments contained ambient seawater (Fig. 1).

**Table 2:** List of all relevant variables considered for the analysis of the effects of temperature differences on the responsiveness of individual reflexes or behaviours, reflex impairment index and survival (dead/alive after 7 days of monitoring) of European plaice (*P. platessa*)

**Variable**	**Description**	**Type**
*Response variables*		
Body flex (behaviour)	Fish is held outside the water on the palms of two hands (touching each other) with its belly facing up and its head and tail unsupported. Response: actively trying to move head and/or tail towards each other, or actively struggling off the hand.	Binary
Righting (behaviour)	Fish is held underwater at the surface on the palms of two hands (touching each other) with its belly facing up and then is slowly released. Response: actively righting itself under water.	Binary
Head complex (reflex)	The fish is held by its body out of water with its dorsal side facing up, and its head and operculum are observed for 5. The head points away from the handler. Response: fish opens and closes its mouth and/or operculum at least once.	
Evasion (behaviour)	Fish is held underwater at the surface in an upright position by supporting its belly with the fingers and holding its back by the thumbs. Then the thumbs are lifted and the fish released, whilst still supporting its belly by the fingers. Response: the fish swims actively away towards the bottom of the container.	Binary
Tail grab (reflex)	The tip of the tail is held gently between thumb and index finger (with attention for the presence of spines). Response: actively struggles free and swims away.	Binary
R index	Mean impairment score of the five assessed reflexes.	Continuous
Post-release survival	Status of a fish after 7 d of monitoring (alive, 0 or dead, 1).	Binary
*Tested explanatory variables*		
Seafloor water temperature	Seawater temperature at the seafloor (°C). This is the ambient temperature to which all trawled fish were naturally acclimatized. Temperature was averaged across all records measured with an H2L logger every 2 min whilst net was towed across seafloor.	Continuous
Treatment water temperature	Seawater temperature of the 10-min exposure treatment (°C)—average of 2 values measured when fish was put in and taken out, representing either the ambient water treatment or the modified water treatment.	Continuous
Treatment air temperature	Air temperature of the 10-min exposure treatment (°C)—average of 2 values measured when fish was put in and taken out, representing either the cold air treatment or the warm air treatment.	Continuous
Treatment combination	Cold air–ambient water, warm air–ambient water, cold air–modified water, warm air–modified water	Categorical
∆T water	Absolute difference (∆) in temperature between seafloor water temperature and treatment water temperature (°C) (either from the ambient or modified water treatment).	Continuous
∆T air	Absolute difference (∆) in temperature between seafloor water temperature and treatment air temperature (°C) (either from the cold or warm air treatment).	Continuous
Type of water temperature shock	Temperature shock was defined if the difference between seafloor water temperature and treatment water temperature exceeded 0°C. A cold shock was defined if the water at the seafloor was warmer than the treatment water, whilst a heat shock was defined if the temperature of the treatment water was warmer than the temperature at the seafloor.	Categorical
Type of air temperature shock	The type of a temperature shock was defined if the difference between seafloor water temperature and treatment air temperature exceeded 0°C. A cold shock was defined if the water at the seafloor was warmer than the treatment air, whilst a heat shock was defined if the temperature of the treatment air was warmer than the temperature at the seafloor.	Categorical
Sorting duration	Time in minutes spent on deck after being emptied from the codend and until being sampled.	Continuous
Total air exposure	Sum of time in minutes of all events in which the fish was exposed to air, including sorting duration, waiting time to undergo air treatment, and handling time during reflex assessment, but excluding the 10 min during the air exposure treatment.	Continuous
Gear deployment duration	Duration in minutes between setting the net in the water and pulling it on deck.	Continuous
TL	Plaice total length measured in cm	Continuous

### Sampling

**Figure 2:**
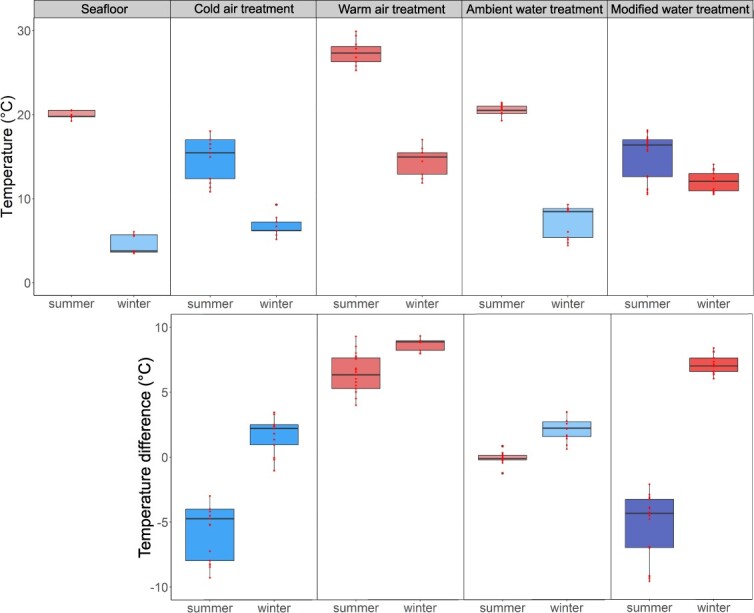
A) Ambient seafloor temperature and treatment air and water temperatures per season^−1^. B) air and water temperature differences with ambient seafloor temperature per season^−1^. Air treatments were in either cold or warm air, whilst water treatments were either in ambient or modified water (cooled in summer, heated in winter). All values given as 10th and 90th percentile with median.

Each batch of 5 plaice was placed into Styrofoam boxes (60 cm L × 40 cm W × 22 cm H) for 10 min as part of the air exposure treatment. Inside the boxes, air was either cooled using 4 conventional ice packs (14.44 ± 0.15°C in summer; 6.33 ± 0.09°C in winter; mean ± SE) or heated using an electric blanket affixed to the lid with duct tape (26.40 ± 0.09°C in summer; 14.04 ± 0.15°C in winter; mean ± SE) (Table 1; Fig. 2). Plaice therefore experienced a difference in air temperature versus ambient seafloor water temperature in summer of −5.57 ± 0.13°C and 6.39 ± 0.10°C (mean ± SE), and in winter −1.88 ± 0.10°C and 9.41 ± 0.11°C (mean ± SE), respectively (Fig. 2B). Whilst the first two batches of fish were exposed to the thermal air treatment, the other two batches were exposed on deck to ambient air temperatures out of direct sunlight. When the first two batches moved to the water treatment, the second ones received the thermal air treatment. The waiting time of the second two batches was included in the total air exposure time to account for the potential influence of this sampling artefact on the results (Table 2). Following the air treatment, each batch of 5 fish was placed into aerated 25 L PVC buckets filled with seawater for 10 min as part of the water treatment. Seawater temperature in the buckets was either at ambient surface water temperature (19.94 ± 0.04°C in summer; 6.88 ± 0.16°C in winter; mean ± SE; Table 1) or at a modified temperature, i.e. cooled in summer (14.75 ± 0.19°C; mean ± SE) or heated in winter (11.65 ± 0.10°C; mean ± SE) (Table 1; Fig. 2). This resulted in plaice being exposed to an ambient versus modified water temperature difference of 0.31 ± 0.02°C and −5.27 ± 0.17°C in summer (mean ± SE), and 2.15 ± 0.08°C and 7.08 ± 0.06°C in winter (mean ± SE), respectively (Fig. 2B). The ambient water treatment was sourced from surface seawater pumped through the deck hose. This may have resulted in the slightly warmer surface water temperatures observed in the winter (Table 1, Fig. 2A). The modified water temperature was achieved in summer with a TECO™ cooling unit (TC 30) and in winter by heating with conventional aquarium heaters. The order of the treatment combination (i.e. which batch was assigned to which combination) was alternated amongst deployments. Throughout the experiments, salinity, seafloor temperature, experimental temperature and dissolved oxygen were monitored using a HydroLab™ HL4 probe in the field, and a YSI™ Inc. Pro2030 water quality meter was placed inside all water-filled containers. Although air and water temperature shocks were designed to be >5°C in absolute values and ambient water temperatures were designed to remain <1°C in absolute values, in two instances the design was confounded: despite the use of ice packs, the cold air treatment temperature in winter was on average 1.88 ± 0.10°C higher than the ambient water temperature at the seafloor. Similarly, as surface waters were used for the ambient water treatment, the ambient water treatment in winter was on average 2.15 ± 0.08°C higher than seafloor temperature. However in the latter case, the absolute temperature difference was <2.5°C, which is much smaller than the difference between seafloor temperatures and the modified treatment temperature.

### Vitality and post-release survival assessments

Following the air and water temperature treatments, individual fish were assessed for reflex behaviour impairment, total length (TL) measured to the nearest centimetre and t-bar anchor tagged for individual identification (see Uhlmann *et al.,* 2016 for details). Each plaice was handled in its respective treatment waters and scored by the same observer on all trips for absence/presence of the following five reflexes/behaviours: body flex, righting, head complex, evasion and tail grab (description in Table 2). The order of batches assessed was alternated amongst deployments.

A visible (non-)response of a fish to a reflex stimulus was defined as either unimpaired (0) or impaired (1). Weak responses were classified as unimpaired (following Meeremans *et al.,* 2017). The corresponding reflex impairment index was calculated for each fish as the mean impairment score of the 5 assessed reflexes; this ranged between 0 (no impairment) and 1 (fully impaired). All vitality-tested plaice were first kept in water-filled 30 L monitoring containers (60 cm L × 40 cm W × 12 cm H; see Uhlmann *et al.,* 2016 for details) aboard the vessel for <5 h and then transported in a truck for maximum 20 min to the laboratory where they were housed in 124 L monitoring containers for 7 days to monitor all treatment-induced effects (ICES, 2014). The water temperature in the monitoring containers was matched as closely as possible to the ambient seafloor water temperature (Table 1). Plaice were fed *ad libitum* defrosted brown shrimp (*Crangon crangon*) throughout the monitoring period, and mortality was assessed daily. The status of each fish was scored as alive (0) or dead (1) daily for 7 days.

### Statistical analysis

Generalized linear mixed models (GLMM) and linear mixed models (LMM) (*glmer* and *lmer* functions from package *lme4,* respectively; Bates *et al.*, 2015) were used to identify parameters that would significantly affect the impairment of each reflex behaviour (binary, present [0] or absent [1]; GLMM), the reflex impairment index (continuous, between 0 [no impairment] and 1 [fully impaired]; LMM) and post-release survival (binary, alive [0] or dead [1]; GLMM). All statistical analyses were performed in R (version 4.1.1; R Core Team, 2022). Prior to any significance testing, all data were explored following the protocol by Zuur *et al.* (2010) checking for outliers, contrasts in the explanatory variables and any correlative relationships amongst all variables. Deployment number and trip number were used as random effects to account for the variability derived from the nested experimental design. First, univariable models were applied for each reflex, the reflex impairment index and survival as a function of each explanatory variable (Table 2 for list and description). For post-release survival, the reflex impairment index was included in the list of tested explanatory variables. The Benjamini–Hochberg procedure with a false discovery rate of 5% was used to control for false-positive results (Benjamini and Hochberg, 1995). The significant, non-collinear variables and, if applicable, their biologically plausible one-way interactions from the univariable analysis were considered for building candidate, multi-variable models for each reflex, the reflex impairment index and survival in all their possible combinations following an information theory approach (Burnham and Anderson, 2004). For each response variable, most parsimonious models were the ones with the lowest Akaike Information Criterion (AIC; Akaike, 1973) value and ranked based on their highest Akaike weights (AICωi; Burnham and Anderson, 2004). Competing models with AICωi ≥ 0.05 were considered (Supplementary Material, Table S1). Models with higher Akaike weights have more empirical support. Best models for each reflex and post-release survival (binary) were validated by k-fold cross-validation, where k represents the number of folds into which the dataset was split (here k = 3). The relationship between occurrence data and the explanatory variables was modelled by using a training dataset (2/3 of the data), and the quality of predictions was assessed using a testing dataset for validation (1/3 of the data). The area under the curve (AUC) of the receiver operating characteristics (ROC) curve was used as a metric of model performance. The AUC measures the ability of the model to correctly predict presences and absences. The sensitivity value measures the percentage of true positives correctly predicted, whilst the specificity value measures the percentage of true negatives correctly predicted (Kleinbaum and Klein, 2010). The best model for the reflex impairment index (a continuous variable) was validated by visually assessing the residuals normality (QQ-plot), the residuals versus fitted values and the residuals versus each covariate.

## Results

A total of 324 plaice (TL: 17.5 ± 0.3 cm, mean ± SE) were sampled, exposed to temperature treatments and assessed for reflex impairment and post-release survival (monitored in captivity for 7 days). Of these, 196 and 128 plaice were sampled in summer and winter, respectively. Plaice sampled in the winter were significantly larger in comparison to the summer sample (LMM, *P* < 0.05). Sorting and total air exposure durations ranged between 6.33 ± 1.26 min (mean ± SD) and 16.00 ± 2.18 min, and 11.16 ± 6.25 and 16.67 ± 13.76, respectively (Table 1).

Seafloor temperature was correlated with air treatment temperature (Spearman’s rank correlation coefficient R^2^ = 0.67) and water treatment temperature (R^2^ = 0.79). Both air temperature difference (∆T air) and water temperature difference (∆T water) were collinear with seafloor water temperature and season (both *P* < 0.001; linear regression, LM). As a consequence of the design, the type of water temperature shock (cold vs. heat) was collinear with seafloor water temperature (R^2^ = 0.78). All reflexes were collinear: if one reflex was impaired, another was more likely to be impaired as well.

**Figure 3:**
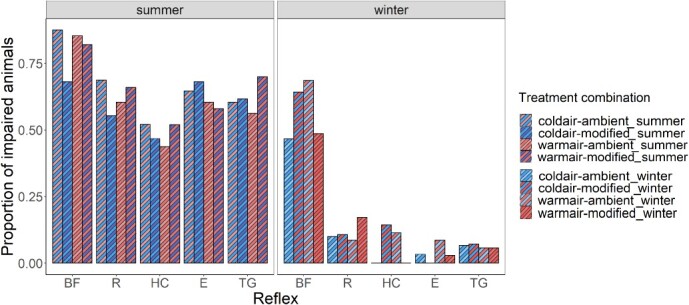
Proportion of impaired animals for each of the 5 reflex responses assessed in sampled plaice (*P. platessa*) after exposure to each treatment combination per season (BF = body flex; R = righting; HC = head complex; E = evasion; TG = tail grab).

Similar trends in the percentage of impairment in fish were noted across all five reflexes: individual reflexes were predominantly impaired when fish were trawled in summer (righting 63%, head complex 49%, evasion 63%, tail grab 62%), compared to winter (righting 12%, head complex 1%, evasion 0%, tail grab 1%), except for body flex, which was frequently impaired in both seasons (81% and 57% in summer and winter, respectively; Fig. 3).

Season significantly influenced the variability in impairment levels of all reflexes, the reflex impairment index and survival across all univariable models, with summer being associated with higher levels of impairment (Table 3). To a lesser degree, sorting duration, total air exposure and TL were relevant. Because season, type of water shock and type of air shock were collinear, these factors were tested in separate candidate models.

**Table 3:** Chi-squared values, significance levels and AIC scores of each relevant explanatory variable (after Benjamini-Hochberg correction, with a false discovery rate of 0.05) tested for each reflex (body flex, righting, head complex, evasion and tail grab), reflex impairment index (R index) and survival using univariable (generalized) linear mixed regression models (GLMM, LMM and GLMM, respectively)

**Variable**	**Body flex**			**Righting**			**Head complex**		
	Chi-sq	*P*-value	AIC	Chi-sq	*P*-value	AIC	Chi-sq	*P*-value	AIC			
**Season**	12.59	**0.00** ^***^	367.0	n/a	n/a	n/a	31.5	**0.00** ^***^	326.7			
**ΔT water**	0.83	0.361	376.0	0.46	0.50	343.3	0.00	0.99	349.8			
**ΔT air**	0.11	0.738	376.7	0.87	0.35	342.8	0.65	0.42	349.2			
**Treatment combination**	8.5	**0.037** ^*^	372.3	2.16	0.54	345.6	4.95	0.18	349.0			
**Type water shock**	9.54	**0.002** ^**^	366.9	15.18	**0.00** ^***^	328.1	n/a	n/a	n/a			
**Type air shock**	8.91	**0.003** ^**^	367.6	1.23	0.27	342.5	0.23	0.63	349.6			
**Gear deployment duration**	0.31	0.581	376.5	1.69	0.19	342.1	0.97	0.32	348.9			
**Sorting duration**	2	0.157	375.0	1.37	0.24	342.4	3.74	0.05	346.2			
**Total air exposure**	0.55	0.461	376.3	3.26	0.07	340.5	5.17	**0.02** ^*^	344.7			
**TL**	6.6	**0.01** ^*^	370.3	0.46	0.50	343.2	11.43	**0.00** ^***^	337.7			
	**Evasion**			**Tail grab**			**R index**			**Survival**		
	Chi-sq	*P*-value	AIC	Chi-sq	*P*-value	AIC	Chi-sq	*P*-value	AIC	Chi-sq	*P*-value	AIC
**Season**	55.18	**0.00** ^***^	302.7	47.85	**0.00** ^***^	317.5	50.60	**0.00** ***	305.4	25.60	**0.00** ^***^	177.2
**ΔT water**	0.32	0.57	339.7	0.03	0.86	348.3	0.01	0.91	346.1	2.29	0.13	203.2
**ΔT air**	0.06	0.81	340.0	0.29	0.59	347.9	0.30	0.59	345.9	0.08	0.77	205.5
**Treatment combination**	0.02	0.90	340.0	1.99	0.57	350.3	3.83	0.28	346.2	4.64	0.20	204.8
**Type water shock**	n/a	n/a	n/a	14.71	**0.00** ^***^	334.6	18.82	**0.00** ***	329.2	2.12	0.15	203.3
**Type air shock**	0.02	0.90	340.0	0.77	0.38	347.4	2.04	0.15	344.1	0.54	0.46	205.1
**Gear deployment duration**	0.92	0.34	339.1	2.21	0.14	346.1	2.05	0.15	344.2	0.73	0.39	204.8
**Sorting duration**	6.51	**0.01** ^*^	333.8	2.44	0.12	345.9	2.99	0.08	343.3	6.59	**0.01** ^*^	196.9
**Total air exposure**	3.73	0.05	336.4	9.28	**0.00** **	338.9	6.98	0.01**	338.9	3.65	0.06	201.8
**TL**	4.75	0.03^*^	335.3	1.58	0.21	346.7	1.87	0.17	344.3	7.72	**0.01** *	197.0

° *P* > 0.05;

^*^ *P* < 0.05;

^**^ *P* < 0.01;

^***^ *P* < 0.001

Season was used as a categorical proxy for ambient seafloor temperature. See Table 2 for variable descriptions. Some models did not converge = n/a.

For body flex, season, type of water and air shock, treatment combination and TL were significantly correlated with impairment (Table 3). Nine candidate models were built for the analysis of the impairment to body flex (only models with AICωi > 0.05 are shown in Table 4); of these the most parsimonious model included type of air shock, treatment combination and TL, with smaller individuals that were exposed to cold air shock followed by a cold water shock showing higher levels of impairment (GLMM, *P* < 0.001, AIC: 357.64; AUC: 0.68; Table 5). For righting, type of water shock was the only significant non-collinear variable that could be considered for model building, with higher impairment after exposure to cold water shocks (GLMM, *P* < 0.05, AIC: 328.10; AUC: 0.69; Table 4 and 5). For head complex, 10 candidate models were built including the significant explanatory factors of season, type of water shock, total air exposure and TL (Table 4). The most parsimonious model included TL, season and total air exposure, with smaller fish captured in summer and with longer air exposure showing higher levels of impairment (GLMM, *P* < 0.05, AIC: 316.01; AUC: 0.79; Table 5). For evasion, 10 candidate models were built including season, TL and sorting duration (Table 4). The most parsimonious model included season and TL as significant fixed effects, with smaller fish captured in summer showing higher levels of impairment (GLMM, *P* < 0.05, AIC: 301.23; AUC: 0.80; Table 5). For tail grab, 5 candidate models were considered that included the significant explanatory factors of season, type of water shock and total air exposure (Table 4). The most parsimonious of these included season and total air exposure, with fish captured in summer and with longer air exposure showing higher levels of impairment (GLMM, *P* < 0.05, AIC: 311.46; AUC: 0.80; Table 5).

**Table 4:** Results of candidate multivariable (generalized) linear mixed models for each reflex tested, the reflex impairment index (R index) and the post-release survival (GLMM, LMM and GLMM, respectively) listing the AIC score, the Δi AIC and the corresponding Akaike weights (AIC_ωi_ ≥ 0.05). Models with higher Akaike weights have more empirical support

**Model**	**AIC**	**Δ**_**i**_ **AIC**	**AIC*ω***_***i***_
*Body flex*			
M1 < body flex ~ TL + treatment combination + type air shock + (1|unique_trawlID)	357.64	0.00	0.52
M2 < body flex ~ TL + type air shock + (1|unique_trawlID)	358.34	0.71	0.37
*Righting*			
M3 < righting ~ type water shock + (1|unique_trawlID)	328.10	0.00	1
*Head complex*			
M4 < head complex ~ TL + season + total air exposure + (1|unique_trawlID)	316.01	0.00	0.52
M5 < head complex ~ TL:season + total air exposure+ (1|unique_trawlID)	317.54	1.53	0.24
M6 < head complex ~ TL + season + (1|unique_trawlID)	318.42	2.41	0.16
M7 < head complex~ TL:season + (1|unique_trawlID)	319.80	3.79	0.08
*Evasion*			
M8 < evasion ~ season + TL + (1|unique_trawlID))	301.23	0.00	0.47
M9 < evasion ~ season + (1|unique_trawlID))	302.65	1.42	0.23
M10 < evasion ~ season + sorting duration + TL+ (1|unique_trawlID))	302.91	1.69	0.20
M11 < evasion ~ season + sorting duration + (1|unique_trawlID))	304.46	3.23	0.09
*Tail grab*			
M12 < tail grab ~ season + total air exposure + (1|unique_trawlID))	311.46	0.00	0.95
*R index*			
M13 < R index ~ season + total air exposure + (1|unique_trawlID))	143.15	0.00	0.67
M14 < R index ~ season + (1|unique_trawlID))	145.40	2.24	0.22
M15 < R index ~ season:total air exposure + (1|unique_trawlID))	146.61	3.45	0.12
*Post-release survival*			
M16 < survival ~ season + sorting duration +(1|unique_trawlID))	176.30	0.00	0.60
M17 < survival ~ season + (1|unique_trawlID)	177.16	0.87	0.39
M18 < summer survival ~ R index + TL + (1|unique_trawlID)	117.97	0.00	0.69
M19 < summer survival ~ R index:TL + (1|unique_trawlID)	121.22	3.25	0.14
M20 < summer survival ~ TL:total air exposure + (1|unique_trawlID)	123.02	5.05	0.06

The list of fixed effects and their interactions tested in multivariable models reflects the selection of significant explanatory variables identified in Table 3. The random effect used was a unique identifier for each trawl deployment nested in trip.

The mean reflex impairment index observed in winter (0.17 ± 0.01; mean ± SE) was lower than the mean reflex impairment index observed in summer (0.64 ± 0.03; mean ± SE), with 4% and 60% of fish experiencing a mean impairment level >0.5, respectively. When tested individually, the variables that best explained the variability in the mean reflex impairment levels were found to be season, type of water shock and total air exposure (Table 3). The most parsimonious model included season and total air exposure as explanatory variables, with fish captured in summer and with longer air exposure exhibiting higher values of reflex impairment index (LMM, *P* < 0.05, AIC: 143.15; Table 5).

**Table 5:** Best and most parsimonious multivariable mixed-effects models and associated outputs (estimated regression parameters, coefficients, z-values and *P*-values) selected for each reflex (GLMM), reflex impairment index (R index, LMM) and post-release survival (GLMM)

**Response variables**	**Model**	**Term**	**Estimate**	**SE**	**z-value**	***P*-value**	**AIC**
**Body flex**	~ TL + treatment combination + type air shock + (1 | unique_trawlID)	(Intercept)	3.33	0.62	5.34	**0.00** ^***^	357.64
		TL	−7.11	2.63	−2.70	**0.01** ^**^	
		TempExposurecoldair-modified	−0.64	0.42	−1.52	0.13	
		TempExposurewarmair-ambient	−0.17	0.46	−0.37	0.71	
		TempExposurewarmair-modified	0.72	0.47	1.53	0.13	
		Type_airwarm_air shock	−1.51	0.45	−3.36	**0.00** ^***^	
**Righting**	~ type water shock + (1|unique_trawlID)	(Intercept)	0.55	0.50	1.10	0.27	328.10
		Type_warmwater shock	−2.07	0.53	−3.90	**0.00** ^***^	
**Head complex**	~ TL + season + total air exposure + (1|unique_trawlID)	(Intercept)	0.94	0.69	1.36	0.17	316.01
		TL	−10.77	3.46	−3.11	**0.00** ^**^	
		seasonwinter	−2.60	0.52	−5.04	**0.00** ^***^	
		totalairexposure	3.42	1.65	2.07	**0.04** ^*^	
**Evasion**	~ season + TL + (1 | unique_trawlID)	(Intercept)	1.50	0.57	2.64	**0.01** ^**^	301.23
		seasonwinter	−3.63	0.51	−7.10	**0.00** ^***^	
		TL	−5.94	3.27	−1.82	0.07	
**Tail grab**	~ season + total air exposure + (1 | unique_trawlID)	(Intercept)	−0.46	0.43	−1.07	0.28	
		seasonwinter	−3.46	0.52	−6.64	**0.00** ^**^	
		totalairexposure	4.61	1.66	2.77	**0.01** ^**^	
**Post-release survival**	~ season + sorting duration + (1 | unique_trawlID)	(Intercept)	−0.74	1.61	−0.46	0.65	176.3
		seasonwinter	−6.95	1.52	−4.58	**0.00** ^***^	
		sortingduration	14.38	8.50	1.69	0.09°	
**Post-release survival (summer)**	~ R index + TL + (1 | unique_trawlID)	(Intercept)	1.85	1.25	1.49	0.14	117.97
		R index	4.18	0.94	4.43	**0.00** ^***^	
		TL	−11.11	6.07	−1.83	0.08°	
							
			**Estimate**	**SE**	**t-value**	***P*-value**	**AIC**
**R index**	~ season + total air exposure + (1 | unique_trawlID)	(Intercept)	0.54	0.06	9.22		143.15
		seasonwinter	−0.46	0.06	−7.08		
		totalairexposure	0.44	0.18	2.43		

° *P* > 0.05;

^*^ *P* < 0.05;

^**^ *P* < 0.01;

^***^ *P* < 0.001

Best models were selected following stepwise forward model selection based on *P*-values, AIC scores and Akaike weights (AIC_ωi_ ≥ 0.05) identified in the previous step (Table 4). The random effect used was a unique identifier for each trawl deployment nested in trip.

**Figure 4:**
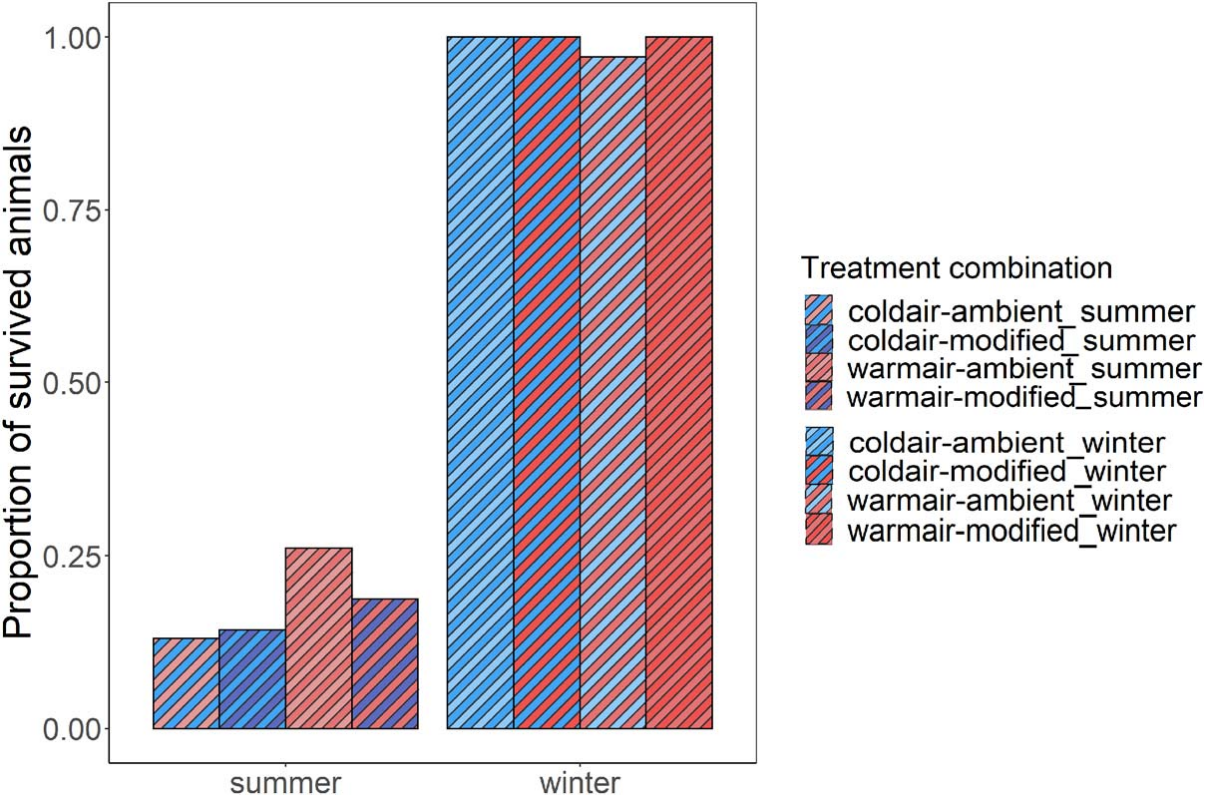
Proportion of sampled and surviving plaice (*P. platessa*) after exposure to each treatment combination per season.

**Figure 5:**
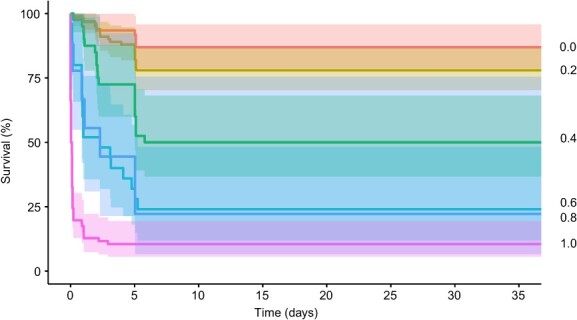
Non-parametric Kaplan–Meier survival probability estimates over days of monitoring of discarded European plaice (*P. platessa*) per reflex impairment index.

Post-release survival was high (99% [95% CI: 97–100%], 127 of 128 fish) in winter, but low (21% [95% CI: 16–28%], 41 of 196 fish) in summer (Fig. 4). Reflex-impaired plaice were more likely to die (with some variability at each impairment interval; Fig. 5). Within summer, mortality was evenly distributed amongst the combination of temperature treatments (χ^2^ = 2.98, df = 3, *P* = 0.39, Bonferroni adjustment, all pairs *P* > 0.05). The variables that best explained the variability in survival probability were found to be season, TL and sorting duration (Table 3). The most parsimonious model included season and sorting duration as explanatory variables, with fish captured in winter subjected to a shorter sorting duration showing a greater probability of survival (GLMM, *P* < 0.05, AIC: 176.30; AUC: 0.90; Table 5).

Given that the reflex impairment index was significantly influenced by the prevailing environmental temperature, its potential correlation with survival was modelled for each season separately. Survival in winter could not be modelled due to the very low mortality (i.e. strongly unbalanced data). In summer, survival was significantly influenced by the seafloor temperature, ΔT air, the reflex impairment index, total air exposure and TL when tested in univariable GLMM models. The most parsimonious multivariable model included the reflex impairment index and TL as explanatory variables, with larger fish with a lower reflex impairment index showing a greater probability of survival (GLMM, *P* < 0.05, AIC: 117.97; AUC: 0.80; Table 5).

## Discussion

This is the first systematic, manipulative experiment to test the effects of temperature on reflex impairment and survival of discarded European plaice. The ambient seafloor water temperature to which fish were acclimatized, as opposed to the temperature differences and exposure to stress during trawling, sorting and discarding, was shown to have the greatest effect on both impairment of reflex behaviours and survival of plaice. In the winter compared to summer, fewer reflexes were impaired and survival was higher amongst beam-trawled plaice, which is in accordance with previous studies that evaluated the effects of temperature on survival of this cold water-adapted species (Van Beek *et al.,* 1990;Uhlmann *et al.,* 2016 ; Uhlmann *et al.,* 2021).

Any physiological changes potentially triggered by two 10-min exposure periods to altered temperature regimes did not result in significant changes of reflex responsiveness, regardless of whether it was a reflex (i.e. head complex, tail grab) or a reflexive behaviour (i.e. evasion), except for the body flex and righting behaviours, which were affected when fish experienced a cold shock. Body flex was affected by type of air shock (cold vs. heat), with higher impairment when plaice were exposed to a cold air shock. Righting was more impaired when exposed to cold water shock (the modified treatment in summer). Sudden decreases in temperature have been associated with loss of dorsoventral orientation in fish (Gale *et al.,* 2011; reviewed in Donaldson *et al.,* 2008; Reid *et al.,* 2022).

It is well established that thermal stress challenges a fish to overcome the effects of capture-and-release events, causing cumulative physiological stress in the short term (e.g. blood parameters; Gale *et al.,* 2013; Prystay *et al.,* 2017) and behavioural impairment in the long term (e.g. loss of equilibrium, swimming impairment, lack of feeding and inability to avoid predation; Gale *et al.,* 2011, 2014; Raby *et al.,* 2014; Pinder *et al.,* 2019). In summer, at 20°C water temperature, the metabolism and energy budget of cold water-adapted plaice is most likely at the upper limit of their thermal tolerance, leading to intolerance to any cumulative stress caused by fishing capture (Uhlmann *et al.,* 2016; Uhlmann *et al.,* 2021). The present study revealed a seasonal effect on true reflexes as well as reflexive behaviours. The head complex reflex was the least affected reflex in both seasons; season, TL and air exposure contributed to the variability in impairment of this reflex. Air exposure was also associated with variability of the tail grab and R index, indicating that prolonged air exposure can have an additive effect that exacerbates impairment due to anaerobiosis (Uhlmann *et al.,* 2016; Methling *et al.,* 2017) which cannot be offset by the capacity of plaice to breathe via their skin (Steffensen *et al.,* 1981).

Overall, body flex was the most sensitive reflex with high levels of impairment in both seasons. Any metabolic stress from the capture process is enough to compromise the contractile ability of white muscle tissue (Methling *et al.,* 2017). Body flex, head complex and evasion were correlated with TL, with smaller fish more likely to experience impairment. This is similar to previous studies (Revill *et al.,* 2013; Uhlmann *et al.,* 2016; Meeremans *et al.,* 2017) but it could be due to a skewed size distribution between seasons acting as a confounding effect, as plaice caught in the winter were marginally but significantly larger than those caught in summer. Smaller fish may have a lower level of physical resilience to fishing capture stress due to lower body mass. However, the opposite has also been proposed, where larger individuals may be more vulnerable to oxygen deficiency due to greater anaerobic energy expenditure compared to smaller conspecifics from increased utilization of ATP and glycogen reserves (Kieffer, 2000; Methling *et al.,* 2017).

Whilst temperatures dictate the rate at which food is metabolized, the size of fish represents the scale at which this occurs (Brett and Groves, 1979). Juvenile plaice thrive in warmer coastal shallows in summer, whereas adults prefer and move to deeper, cooler waters (Fonds *et al.,* 1992; Poos *et al.,* 2013). According to Fonds *et al.,* 1992, thermal optima of plaice shift from 20°C to 10°C as they grow older (and larger), with a shift of 1°C per 10 cm in length (Fonds, pers. comm., in van der Veer *et al.,* 2009). It therefore appears important to consider ontogenetic shifts in thermal physiology and tolerance levels when investigating thermal effects on the survival of landed fish.

After release, the fish need to recover from the severe, acute stress from the fishing process (i.e. capture, sorting, release). Whilst recovering, they are unable to resume basic activities (e.g. feeding; Raby *et al.,* 2014). Our findings revealed that post-release survival was heavily influenced by seasonality, with low survival in summer (21%) compared to winter (99%), regardless of the temperature treatment combination experienced. It is likely that in summer, plaice were intolerant to the cumulative thermal and physical stress, causing irreversible damage. A similar pattern was observed by Savina *et al.* (2019), Kraak *et al.* (2019), Methling *et al.* (2017) and van Der Reijden *et al.* (2017). For the fish monitored in the summer, survival probability was predicted by the reflex impairment index and fish size, both of which were relevant explanatory variables in earlier work (Uhlmann *et al.,* 2016). Sorting duration partially influenced survival, but this may be due to an artefact of sampling, as sorting duration took (not significantly) longer in the summer compared to winter due to the lower proportion of suitably sized fish in the catches. In any case, minimizing air exposure during sorting may be necessary to maximize post-release survival.

This study has some limitations. We evaluated only a tertiary (vitality, mortality/survival) stress response to environmental stressor of temperature (change). Primary (corticosteroids) or secondary (metabolites, osmolytes and acid–base status) stress responses were not measured, which could have been relevant for a more mechanistic understanding of underlying physiological processes (Horodysky *et al.,* 2015). Another limitation of our study was that the modified water treatment only deviated from ambient temperature to the temperature opposite the seasonal average (i.e. cold shock in summer, heat shock in winter) to mirror the potential seasonal scenarios a fish may encounter when caught and released into a thermocline. However, this experimental design introduced a confounding effect that precluded the possibility to distinguish the effect of ambient temperature (season) from the effect of the water temperature treatment and to test for this in the same model. In future experiments, tested temperature combinations should have the same levels within each treatment group to facilitate model interpretation. Based on the results obtained here, fixed shock thresholds may not be appropriate to investigate temperature role in plaice physiological responses given the interplay of several factors including size and the lack of a known thermal performance curve (Lefevre *et al.,* 2021). An absolute temperature difference of >5°C may be already beyond physiological capacity for a fish at the upper limit of its thermal tolerance (Schulte, 2014). Flexible and informed temperature differences would be preferred to model the effect of thermal shocks and should thus be implemented in future experiments. In general, field experiments such as this, which quantify discard survival by monitoring fish in confinement, are challenged by a lack of accurate dose–response relationships measured for individual fish, because, e.g. the time of capture is unknown and so gear deployment durations are proxies for the level of fatigue experienced inside the net. Whilst treatment temperature deviations were chosen to reflect plausible conventional fishing conditions, fishing was done by a research vessel operating under more benign conditions (lighter beam-trawl gear, shorter deployments, smaller catches).

Despite these shortcomings, we conclude that any interseasonal reflex behaviour assessments need to consider temperature when predicting discard survival. Despite its hypersensitivity, the body flex reflex may still be relevant for plaice to be scored to differentiate slightly stressed from unstressed individuals (Uhlmann *et al.,* 2016). Standardization of sampling and scoring protocols is critical to minimize any confounding effects of air exposure.

This study has implications for commercially trawled plaice that are exempt from the European landing obligation to not discard any less than MCRS plaice (STECF, 2023). Arguably, as previous research had indicated, discarding plaice that have gone through a commercial beam-trawl capture event in summer is likely to be fatal (Uhlmann *et al.,* 2016, 2021, 2023). Accordingly, it can be sensible to grant an actionable exemption in those seasons and regions when release survival can be maximized and routinely collect vitality-relevant parameters during at-sea monitoring campaigns (Falco *et al.,* 2022). It is likely that under current climate change scenarios, warming seawater temperatures will only further compromise the reasoning behind the existing high survival exemption for plaice. It is reasonable to assume that prolonged summer seasons will occur with more frequency, leading to near-0% survival of commercially discarded plaice (Uhlmann *et al.,* 2023). A northward shift in distribution seems inevitable for plaice (Townhill *et al.,* 2023). Additionally, given the observed impact of air exposure related to sorting processes on deck on reflex impairment and survival, controlling and reducing air exposure intervals remains critical to maximize survival of discarded fish and to promote animal welfare-conscious fishing in the 21st century.

## Supplementary Material

Web_Material_coae036
